# The Association between the Pro12Ala Variant in the PPARγ2 Gene and Type 2 Diabetes Mellitus and Obesity in a Chinese Population

**DOI:** 10.1371/journal.pone.0071985

**Published:** 2013-08-21

**Authors:** Xia Wang, Jun Liu, Yingying Ouyang, Min Fang, Hui Gao, Liegang Liu

**Affiliations:** 1 Department of Maternal and Child Health Care, School of Public Health, Shandong University, Jinan, China; 2 Department of Nutrition and Food Hygiene and MOE Key Lab of Environment and Health, School of Public Health, Tongji Medical College, Huazhong University of Science and Technology, Wuhan, China; Tulane School of Public Health and Tropical Medicine, United States of America

## Abstract

**Background:**

Conflicting results have been reported on the association of the Pro12Ala polymorphism of the PPARγ2 gene with the risk of type 2 diabetes or obesity.

**Methods and Findings:**

A total of 3146 subjects with 1145 cases of type 2 diabetes and 2001 healthy controls were included in the study. Genomic DNA was obtained from blood samples and the screening for the gene polymorphisms was done using an allelic discrimination assay-by-design TaqMan method. Overall, the Ala allele frequency was 5.6% in control subjects and 3.9% in diabetes subjects (*P = *0.023). We found a statistically significant association of carriers of the Ala allele with greater homoeostasis model assessment of beta cell function index in all subjects (*P = *0.046). After controlling for confounders, carriers of the Ala allele had a decreased risk of diabetes compared with noncarriers [odds ratio (OR) 0.64, 95% confidence interval (CI) 0.49–0.83; *P = *0.001]. A beneficial effect of the Ala allele was also observed for obesity (OR 0.64, 95% CI 0.42–0.96; *P = *0.030).

**Conclusion:**

Our results suggested that the presence of the Ala allele may contribute to improved insulin secretory capacity and may confer protection from type 2 diabetes and obesity in the Chinese population.

## Introduction

Obesity and type 2 diabetes mellitus (T2DM) are major worldwide public health problems [1], [2]. Although China was once thought to have one of the leanest populations, it is fast catching up with the West in the prevalence of obesity and diabetes. This transition has occurred in a remarkably short time with a rapid change in lifestyle in China [3]. Between 1992 and 2002, the Chinese obesity standard presents an increase from 20.0 to 29.9% [4]. Obesity is significantly related to the incidence of diabetes.

The peroxisome proliferator-activated receptor γ2 (PPARγ2) is a nuclear receptor transcription factor involved in lipid metabolism, adipocytes differentiation and proliferation, and insulin sensitivity by regulating the expression of adipocyte-specific developmental genes [5]. The PPARγ2 Pro12Ala polymorphism, which is the result of a CCA-to-GCA missense mutation in codon 12 of exon B, encodes the amino-terminal domain defining the adipocyte specific PPARγ2 isoform of the PPARγ gene [6]. The association between the Pro12Ala variant in the PPARγ2 gene and T2DM or obesity have been the most extensively examined in Caucasians [7]–[12]. However, the Ala frequencies have difference among different ethnic groups. Although several studies [13]–[18] have described the associations with T2DM or obesity risk in the Chinese population, they involved small samples (fewer than 208 cases) and did not consider the impact of important confounding factors on the associations. Further, these studies yielded conflicting results. In addition, it has been postulated that PPARγ may have an effect on lipid metabolism, insulin resistance, and insulin secretion by inducing the transcription of target genes [7], [19]–[21]. However, results from studies investigating the association between PPARγ2 variants and these variables in the Chinese population are inconsistent and also scarcer.

Therefore, the purpose of this study was to evaluate the association of the Pro12Ala polymorphism of the PPARγ2 gene with plasma lipids, insulin resistance, and insulin secretion in a relatively large Chinese population and to examine whether the Pro12Ala polymorphism was associated with the risk of T2DM and obesity in this population.

## Methods

### Study Population

The study population consisted of 1145 newly diagnosed T2DM patients and 2001 healthy controls with normal glucose tolerance (NGT). Subjects were consecutively recruited from patients attending the outpatient clinics of the Department of Endocrinology, Tongji Hospital, Wuhan, China. Controls were recruited from an unselected population undergoing a routine health check-up at the same hospital from December 2004 to November 2007. T2DM patients met the well-established diagnostic criteria recommended by American diabetes association and World Health Organization.

All participants, including their parents and grandparents, were from the Chinese Han population. Personal information on demography was collected by using a medical health questionnaire, including sex, age, height, weight and blood pressure. Body mass index (BMI) was calculated as weight divided by the square of height (kg/m^2^). Family history of diabetes was positive if first-degree relatives (parents, siblings) had T2DM.

### Laboratory Measurements

After a 10 h overnight fast, all participants underwent a 75 g oral glucose tolerance test and venous blood samples were collected at 0 and 2 h for determination of fasting plasma glucose, 2 h oral glucose tolerance test, fasting plasma insulin, total cholesterol, triglyceride, homoeostasis model assessment of insulin resistance (HOMA-IR), homoeostasis model assessment of beta cell function (HOMA-beta), and high-density lipoprotein cholesterol (HDL-C) as described in our previous study [22]. Participants gave informed written consent to the study and did not take any medication known to affect glucose tolerance or insulin secretion before laboratory tests. The study was approved by the Ethics Committee of the Tongji Medical College.

### Genotyping

Fasting venous blood was collected in 5 ml EDTA tubes, and genomic DNA was isolated with standard phenol/chloroform-based method [23]. The genotyping of the Pro12Ala polymorphism rs1801282 was done using an allelic discrimination assay-by-design TaqMan method on ABI7900HT (Applied Biosystems, Foster City, CA, USA). The TaqMan Assay kit was purchased from Applied Biosystems, which included the forward target-specific polymerase chain reaction primer, the reverse primer, and the TaqMan MGB probes labeled with two special dyes: FAM and VIC.

The TaqMan genotyping reaction was amplified (50°C for 2 min, 95°C for 10 min, followed by 40 cycles of 92°C for 15 s and 60°C for 1 min), and the endpoint fluorescent readings were performed by ABI 7900HT data collection. Automatic allele calling, with the default settings, was carried out by ABI7900HT data collection and analysis software version 2.2.1 (SDS 2.2.1).

### Statistical Analysis

Continuous variables are reported as the mean ± SD. Data with a normal distribution were compared by Student t test, and those with unequal variance or without a normal distribution were analyzed by a Mann-Whitney rank sum test. Categorical values were compared by a Chi-square test, which was also used to test for deviation of genotype distribution from Hardy-Weinberg equilibrium. The association between the Pro12Ala polymorphism and T2DM risk was estimated by calculating odds ratios (ORs) and 95% confidence intervals (CIs) from the multivariate logistic regression analyses. An unconditional logistic model was used to adjust for multiple cardiovascular risk factors, such as sex, age, BMI, hypertension, and family history of diabetes. The significance of multiplicative interactions between the single nucleotide polymorphisms and covariates was determined by the logistic regression model.

All study subjects were stratified based on BMI according to the recommendations of Working Group on Obesity in China [24]–[26]. A BMI of 18.5 to 23.9 is considered optimal, 24.0 to 27.9 is overweight, and 28.0 and above is obese.

All tests were two-sided and *P* values<0.05 were considered statistically significant. All data analyses were carried out with using SPSS for windows software version 20.0 (SPSS Inc, Chicago, IL, USA).

## Results

### General Characteristics of the Study Populations

The general characteristics of T2DM and control subjects are presented in Table 1. Compared to NGT subjects, the T2DM subjects were older, and clinical parameters such as BMI (T2DM 25.1±3.9 vs. NGT 23.4±3.7 kg/m^2^, *P*<0.001), fasting plasma glucose (T2DM 9.8±3.0 vs. NGT 4.8±0.7 mmol/L, *P*<0.001), 2 h oral glucose tolerance test (T2DM 16.5±4.8 vs. NGT 6.4±1.1 mmol/l, *P*<0.001), total cholesterol, triglycerides, and the percentage of subjects with hypertension and family history of diabetes were significantly higher in cases (T2DM) compared to controls (NGT). Also, the T2DM subjects had a significantly higher HOMA-IR (*P*<0.001) but a lower HOMA-beta (*P*<0.001) compared to controls. Additionally, no significant differences (*P*>0.05) were observed for sex, fasting plasma insulin and HDL-C levels in T2DM subjects compared to controls (*P*>0.05).

**Table 1:** Clinical and Biochemical Characteristics of the Study population.

Variable	T2DM (n = 1145)	NGT (n = 2001)	*P*
Age (years)	50.5±11.0	43.5±11.6	<0.001
Gender (female/male) (%)	44.1/55.9	41.2/58.8	0.116
BMI (kg/m^2^)	25.1±3.9	23.4±3.7	<0.001
Fasting plasma glucose (mmol/l)	9.8±3.0	4.8±0.7	<0.001
2-h OGTT (mmol/l)	16.5±4.8	6.4±1.1	<0.001
Fasting plasma insulin (µU/ml)	10.9±11.0	9.9±6.6	0.237
Family history of diabetes (no/yes) (%)	80.2/19.8	85.7/14.3	<0.001
Hypertension (no/yes) (%)	56.2/43.8	76.6/23.4	<0.001
HOMA-beta^a^	46.9±71.8	113.4±65.2	<0.001
HOMA-IR^a^	4.4±4.4	2.4±1.3	<0.001
HDL-C (mmol/l)	1.5±0.9	1.4±0.6	0.176
Total cholesterol (mmol/l)	4.7±1.3	4.4±0.9	<0.001
Triglyceride (mmol/l)	2.1±1.6	1.5±1.0	<0.001

Data are mean ± SD values.

Abbreviations: BMI, body mass index; HDL-C, high density lipoprotein cholesterol; HOMA-beta, homeostasis model assessment of beta cell function index; HOMA-IR, homeostasis model assessment of insulin resistance index; NGT, normal-glucose tolerant; OGTT, oral glucose tolerance test; T2DM, type 2 diabetes mellitus.

a Calculated using fasting plasma glucose (mmol/L) and fasting plasma insulin (mU/L) levels.

### Association of the PPARγ2 Pro12Ala Polymorphism with T2DM

Table 2 presents the genotype and allele frequencies of the Pro12Ala polymorphism screened in the study population. The genotype frequencies of the PPARγ2 Pro12Ala variants in subjects with T2DM and NGT subjects were in agreement with the Hardy-Weinberg equilibrium.

**Table 2:** Association of the Pro12Ala polymorphism with type 2 diabetes mellitus.

Allele and genotype	D2TM (%)	Controls (%)	Crude OR (95% CI), *P*	Adjusted OR (95% CI), *P*
Pro/Pro	1060 (92.6)	1783 (89.1)	1.00	1.00
Pro/Ala and Ala/Ala	85 (7.4)	218 (10.9)	0.63 (0.48–0.81), 0.001	0.64 (0.49–0.83), 0.001
Pro	2201 (96.1)	3779 (94.4)	1.00	1.00
Ala	89 (3.9)	223 (5.6)	0.67 (0.52–0.86), 0.002	0.69 (0.54–0.88), 0.003

Adjusted for age, sex, BMI, hypertension, and family history of diabetes.

Abbreviations: CI, confidence interval; OR, odds ratio. The other abbreviations used see Table 1.

As shown in Table 2, there were significant differences in the allelic frequency of the Ala12 variant between T2DM cases and controls. The frequency of the Ala allele was significantly lower in T2DM subjects (3.9%) compared to NGT subjects (5.6%) (*P = *0.023).

The Ala allele had a significant protective association (OR 0.67; 95% CI 0.52 to 0.86, *P* = 0.002). In addition, a decreased OR for T2DM was observed in carriers of the Ala allele (Pro12Ala or Ala12Ala) as compared with those with Pro12Pro genotype (OR = 0.63, 95% CI 0.48 to 0.81, *P* = 0.001). This association remained statistically significant (*P* = 0.001) even after additional adjustment for age, sex, BMI, hypertension, and family history of diabetes (OR = 0.64, 95% CI 0.49 to 0.83) (Table 2).

The comparison of the clinical profiles between Ala carriers and the homozygous normal genotype with and without diabetes are shown in Table 3. No statistically significant associations with plasma lipids were observed for this polymorphism in every stratum. However, in all subjects, there were statistically significant differences in HOMA-beta values, with carriers of the Ala12 allele having higher HOMA-beta values than Pro12 homozygotes (97.3±65.8 vs. 88.3±75.7, *P* = 0.046). In the T2DM subjects, no statistically significant increase in HOMA-beta values related to the Ala allele was observed (49.2±60.6 vs. 46.5±72.6, *P* = 0.749). In the controls, the presence of the Ala allele was not associated with HOMA-IR, HOMA-beta, HDL-C, total cholesterol, or triglyceride. However, in all groups, subjects with Ala allele had lower levels of BMI and in the groups of all subjects and controls, this relationship was statistically significant (*P = *0.006 and *P = *0.040, respectively).

**Table 3:** Comparison of clinical characteristics between subjects with and without the Pro12Ala variant of PPARγ2.

Variable	All Subjects			T2DM			NGT		
	Pro/Ala and Ala/Ala	Pro/Pro	*P*	Pro/Ala and Ala/Ala	Pro/Pro	*P*	Pro/Ala and Ala/Ala	Pro/Pro	*P*
Number	303	2843	–	85	1060	–	218	1783	–
Age (years)	45.7±11.1	46.0±11.9	0.623	51.4±10.7	50.4±11.0	0.435	43.4±10.4	43.5±11.7	0.976
BMI (kg/m^2^)	23.4±3.8	24.1±3.8	0.006^b^	24.8±4.0	25.1±3.9	0.472	22.9±3.6	23.4±3.7	0.040^b^
HOMA-IR^a^	2.9±2.1	3.2±3.1	0.152	4.1±3.2	4.4±4.5	0.494	2.4±1.1	2.4±1.3	0.774
HOMA-beta^a^	97.3±65.8	88.3±75.7	0.046^b^	49.2±60.6	46.5±72.6	0.749	116.1±57.8	113.1±66.0	0.524
HDL-C (mmol/L)	1.5±0.6	1.4±0.7	0.573	1.6±1.0	1.5±0.9	0.192	1.4±0.4	1.4±0.6	0.837
Total cholesterol (mmol/L)	4.5±1.1	4.5±1.1	0.837	4.8±1.4	4.7±1.3	0.596	4.4±1.0	4.4±0.9	0.823
Triglyceride (mmol/L)	1.7±1.2	1.7±1.3	0.756	2.1±2.1	2.1±1.6	0.745	1.5±0.9	1.5±1.1	0.843

All data are means±SD.

The abbreviations used see Table 1 and 2.

a Calculated using fasting plasma glucose (mmol/L) and fasting plasma insulin (mU/L) levels.

b *P*<0.05.

### Association of Pro12Ala Polymorphism with Obesity

All study subjects were stratified based on BMI according to the recommendations of Working Group on Obesity in China. Table 4 shows the genotype and allele frequencies of the PPARγ2 Pro12Ala variants in the study population stratified by BMI.

**Table 4:** Distribution of allele and genotype frequencies of the Pro12Ala polymorphism in study subjects stratified by BMI.

Subjects	PPAR genotypes		Crude OR (95% CI), *P*	Adjusted OR (95% CI), *P*	Ala allelic frequencies (%)
	Pro/Pro (%)	Pro/Ala and Ala/Ala (%)			
BMI 24 (n = 1772)	1582 (89.3)	190 (10.7)	1.00	1.00	5.5
BMI 24–27.9 (n = 983)	898 (91.4)	85 (8.6)	0.77 (0.58–1.00), 0.051	0.78 (0.59–1.02), 0.072	4.5
BMI ≥28 (n = 391)	363 (92.8)	28 (7.2)	0.61 (0.40–0.93), 0.021	0.64 (0.42–0.96), 0.030	3.8

Adjusted for age, sex, BMI, hypertension, and family history of diabetes.

The abbreviations used see Table 1 and 2.

The frequency of Ala carriers were significantly lower in obese subjects (7.2%) compared to non-obese subjects (10.7%) (*P = *0.044). To evaluate the effect of the genotypes on the disease, logistic regression analysis was conducted. The comparison between the Ala carriers and individuals with the Pro12Pro genotype among obese subjects yielded an unadjusted OR of 0.61, which was statistically significant (*P* = 0.021), the significance remained after adjusting for age, sex, hypertension, and family history of diabetes (OR = 0.64, 95% CI 0.42 to 0.96, *P* = 0.030). Similarly, the frequency of Ala carriers were also lower in overweight subjects (8.6%) compared to non-obese subjects (10.7%), but there was not statistically significantly (*P* = 0.092).

### Association of Pro12Ala Polymorphism with Obesity among the NGT Subjects

Because of potential confounding between T2DM and obesity, we also did a separate analysis to look at the association of the Pro12Ala polymorphism with obesity in NGT subjects. The frequency of the Ala carriers were significantly lower (7.7%) in obese subjects compared with the non-obese subjects (10.5%) (*P* = 0.048). For the overweight subjects, the genotype and allele frequencies of the Pro12Ala polymorphism were not significantly different between the overweight and non-obese subjects.

## Discussion

The present study with a total of 3146 participants, including 1145 cases of T2DM and 2001 healthy controls, demonstrated a significant effect of the Ala allele on improved insulin secretory capacity and lower development of T2DM in the Chinese Han population. The beneficial effect of the Ala allele on obesity was also observed by using BMI cut points of the recommendations of Working Group on Obesity in China.

In this study, the frequency of the Ala allele in participants with and without diabetes were 3.9% and 5.6%, respectively, in a Chinese Han population. The frequency of the Ala allele appears to vary greatly by the genetic background of the populations, ranging from 4% to 55% [27]. In general, the frequency of the Ala allele has been reported to be highest in Caucasians [6]. The samples with the lowest rates included non-Caucasian populations, such as Japanese [7], [28], Korean [29], African [30], and Asian descendants [20].

We found that the Ala allele of the Pro12Ala polymorphism was associated with a significantly lower risk of type 2 diabetes in a Chinese Han population. Consistent with our results, Altshuler et al. [9] found a significant decrease in diabetes risk associated with the Ala allele in a Caucasian population. In Finnish subjects, the Ala12 variant of the PPAR**γ**2 gene was associated with protection against T2DM [12]. Meta-analyses [31], [32] also showed a significant effect of the Ala allele on lower development of T2DM. In addition, in the present study, we also found that subjects with the Ala allele had lower HOMA-IR values, although they did not reach statistical significance. Li et al. [33] reported that the Ala allele was associated with lower levels of insulin and HOMA-IR and attenuated the adverse association of adiposity with insulin resistance measures in an unselected community-based sample of whites. Among glucose-tolerant Swedish Caucasian men, Ek et al. [11] also found that the Ala allele was associated with improved whole body insulin sensitivity. Similarly, Lindi et al. [12] and Deeb et al. [7] observed an association of the Pro12Ala polymorphism with better insulin sensitivity. Also, in obese subjects, Koch et al. [34] indicated that the Pro12Ala polymorphism was associated with improved insulin sensitivity as evaluated by the euglycemic hyperinsulinemic glucose clamp. Insulin resistance is a known risk factor for developing cardiovascular disease and T2DM. It is possible that carriers of the Ala allele may have a decreased risk for developing cardiovascular disease and T2DM. Consistent with this hypothesis, some studies [35]–[38] also showed that the PPARγ2 Ala12 allele contributed to a reduced cardiovascular risk profile or coronary heart disease, in addition to many studies demonstrating the effect of the Ala allele on lower development of T2DM.

The substitution from proline to alanine at codon 12 has been shown to regulate transcriptional activity [7], [39]. Because this polymorphism is next to the amino-terminus of the protein in the ligand-independent activation domain, its activity is induced by insulin through phosphorylation. Alanine helps the formation of α-helices, but proline prevents it. Thus, it is possible that this amino acid change affects the structure and consequently the function of the protein [27]. The alanine isoform contributes to less efficient stimulation of PPARγ target genes and predisposes people to reduce levels of adipose tissue mass accumulation. This in turn may improve insulin sensitivity. It is known that decreased insulin sensitivity plays an important role in the pathogenesis of type 2 diabetes.

In addition, our results showed a statistically significant association of Ala allele carriers with greater HOMA-beta values in all subjects, indicating the beneficial effect of the Ala allele on insulin secretory capacity. The T2DM subjects with the Ala allele also had higher HOMA-beta values, but these did not reach statistical significance. PPARγ is highly expressed at the mRNA and protein levels in human islet endocrine cells [40]. PPARγ overexpression may significantly inhibit insulin secretion caused by stimulatory concentration of glucose in rat [41]. Stefan et al. [42] reported that subjects with the Pro12Ala polymorphism had lower insulin secretion after intralipid infusion compared to control subjects, suggesting that the Pro12Ala polymorphism might be involved in a differential regulation of insulin secretion in humans. This further demonstrates the functional importance of this polymorphism in the pathogenesis of T2DM. However, within the non-diabetic population, many studies have reported that the Pro12Ala polymorphism had no significant effect on diabetes-related traits [43]–[45], which may have been due to low Ala frequency in that population, small number of individuals with the Ala allele, and a lack of statistical power.

Carriers of the Ala12 allele had higher HDL-C and lower triglyceride concentrations than those with the Pro12Pro genotype, but we did not find a statistically significant. In the Japanese population, Mori et al. [46] found that carriers of the Ala12 allele had higher total cholesterol than those without the allele among diabetic subjects. In contrast, studies by Beamer et al. [47] and Swarbrick et al. [48] demonstrated that a trend towards lower levels of HDL-C in the Ala allele carriers compared with subjects with the Pro12Pro genotype. One study in an Italian population [49] found that this polymorphism was not associated with anthropometrical and biochemical parameters among normoglycemic and diabetic subjects. Ethnic differences, study design, and effects of BMI may contribute to discrepancies in these results.

The definitions of obesity recommended by the World Health Organization are based on data from Western populations. A growing body of studies shows that these cutoffs likely need to be lower in the Chinese population. Several studies in Chinese populations indicated that Asians have higher amounts of body fat at lower BMIs than do Western populations [50], [51], which perhaps leads to the greater prevalence of cardiovascular disease risk factors at lower BMIs in Chinese populations compared with Western populations [25]. Although one study [13] explored the relationship between the Pro12Ala polymorphism in the PPARγ2 gene and obesity in Chinese population, this study involved small samples (fewer than 160 cases) and did not report ORs of obesity and the corresponding 95% CIs. However, all other studies on the association between the Pro12Ala mutation and BMI or obesity in Asian populations were based on the World Health Organization BMI cut points. Therefore, in accordance with the BMI cut points of the recommendations of Working Group on Obesity in China, the present study examined the association of the Pro12Ala polymorphism in the PPARγ2 gene with obesity. Our results showed that the Pro12Ala polymorphism was associated with obesity in the Chinese Han population. The results supported the hypothesis that the Pro12Ala polymorphism had a marked effect on BMI in apparent obese individuals [52]. The study by Masud et al. demonstrated that a mean BMI<27 kg/m^2^ was not associated with the Pro12Ala polymorphism, whereas a mean BMI>27 kg/m^2^ showed such an association [53]. The expression of the PPARγ gene is increased in the adipose tissue of obese subjects and a low-calorie diet can down-regulate its expression [53], [54]. Thus, the pathogenesis of obesity is probably related to a large number of genetic and environmental factors.

Although this study includes a relatively large simple size, it has limitations. We have adjusted for some confounders, for example, age, sex, BMI, hypertension and family history of diabetes. However, we cannot rule out that uncontrolled or unmeasured risk factors, such as history of coronary heart disease in baseline, that may potentially confound the associations between the Pro12Ala polymorphism and T2DM and obesity.

In conclusion, our results suggested that the presence of the Ala allele may contribute to improved insulin secretory capacity and may confer protection from T2DM and obesity in the Chinese population.
